# Supplementary material for: Is the Social Gradient in Net Survival Observed in France the Result of Inequalities in Cancer-Specific Mortality or Inequalities in General Mortality?
Source: Cancers (Basel). 2023 Jan 20;15(3):659. doi: 10.3390/cancers15030659 (PMC9913401; doi:10.3390/cancers15030659)
Supplement: Supplementary file 1 [file cancers-15-00659-s001.zip › Supplementary Information.pdf]

Supplementary Information

Detailed calculation process for the construction of the simulated deprivation-specific French life tables used in the analyses.

For the present study, in order to re-analyze cancer registries data from Tron et al.,[1] deprivation-specific mortality rates in the simulated life tables (LT) were needed for each stratum of sex (males and females), age (0 to 99, 1-year units), year (2006 to 2013, 1-year units), and the 17 French *Département* (noted "dept" in the following formulas) covered by a cancer registry included in the present study (Calvados, Côte d'Or, Doubs, Finistère, Gironde, Hérault, Isère, Loire-Atlantique, Manche, Bas-Rhin, Haut-Rhin, Saône et Loire, Somme, Tarn, Vendée, Haute-Vienne, Lille area, cf. table 1 in Tron et al.,[1]).

In the England LT, data were available only until 2011, therefore data relative to year 2011 were duplicated for 2012 and 2013. In the Permanent Demographic Sample, *Echantillon Démographique Permanent* (EDP), data were available only for the period 2012-2016, therefore data relative to that period were duplicated for years 2006 to 2013 (thus considering constant social gradient of mortality over time). Similarly, data from external sources (by sex, age, year) were duplicated for each French *Département,* assuming constant social gradient of mortality from one French *Département* to another.

First, we consider the relation (1) in the original French LT :

$(1)\ \boldsymbol{\lambda}(\text{sex, age, year, dept}) = \sum_{i=1}^{5} \mathbf{p_i}(\text{dept})\boldsymbol{\lambda}_i(\text{sex, age, year, dept})$, with λ(sex, age, year, dept) the mortality hazard function, *i* the deprivation category (i=1 to 5 deprivation categories), p_i the proportion of deprivation i, in the general population according to the French *Département*, λ_i(sex, age, year, dept) the mortality hazard function of deprivation *i*.

Then considering rate ratios RR^*_i between the deprivation categories *i* and the reference (i.e. all categories *i* combined), provided by external sources (respectively Eng LT and EDP) noted *, one can define the relation (2):

$(2)\ \boldsymbol{\lambda^*_i}(\text{sex, age, year, dept}) = \boldsymbol{\lambda^*_{ref}}(\text{sex, age, year, dept})\mathbf{RR^*_i}(\text{sex, age, year, dept})$, with λ^*_i(sex, age, year, dept) the mortality hazard function of deprivation *i* provided by external sources, λ^*_{ref}(sex, age, year, dept) the reference mortality hazard function (i.e. all categories *i* combined), provided by external sources (RR^*_{ref}(age)=1).

And also, in the same way, in the original French LT, we have the following relation:

$(2')\ \boldsymbol{\lambda_i}(\text{sex, age, year, dept}) = \boldsymbol{\lambda_{ref}}(\text{sex, age, year, dept})\mathbf{RR_i}(\text{sex, age, year, dept})$

Based on (1) and (2'), in the original French LT, we have:

$(3)\ \boldsymbol{\lambda}(\text{sex, age, year, dept}) = \sum_{i=1}^{5} \mathbf{p_i}(\text{dept})\boldsymbol{\lambda_{ref}}(\text{sex, age, year, dept})\mathbf{RR_i}(\text{sex, age, year, dept})$

We now assume that RR_i(sex, age, year, dept) in the original French LT can be replaced by RR^*_i(sex, age, year, dept) from the external source, giving the following relation:

$(3')\ \boldsymbol{\lambda}(\text{sex, age, year, dept}) = \sum_{i=1}^{5} \mathbf{p_i}(\text{dept})\ \boldsymbol{\lambda}_{\mathbf{ref}}(\text{sex, age, year, dept})\mathbf{RR^*_i}(\text{sex, age, year, dept})$**,** and so:

$\boldsymbol{\lambda}(\text{sex, age, year, dept}) = \boldsymbol{\lambda}_{\mathbf{ref}}(\text{sex, age, year, dept}) \sum_{i=1}^{5} \mathbf{p_i}(\text{dept})\mathbf{RR^*_i}(\text{sex, age, year, dept})$, and also:

$$\boldsymbol{\lambda}_{\mathbf{ref}}(\text{sex, age, year, dept}) = \frac{\boldsymbol{\lambda}(\text{sex,age,year,dept})}{\sum_{i=1}^{5} \mathbf{p_i}(\text{dept})\mathbf{RR^*_i}(\text{sex,age,year,dept})}$$

From that last equation, we can directly estimate λ_{ref}(sex, age, year, dept) since λ(sex, age, year, dept) is known from for the original French LT, p_i(dept) is known from previous work on EDI development[2,3] and RR^*_i (sex, age, year, dept) is obtained from the external sources.

Then, because of (2') and the hypothesis that RR_i(sex, age, year, dept)=RR^*_i(sex, age, year, dept), in a final step we can calculate the λ_i(sex, age, year, dept) for each deprivation category *i*, as follows:

$\boldsymbol{\lambda}_1(\text{sex, age, year, dept}) = \boldsymbol{\lambda}_{\mathbf{ref}}(\text{sex, age, year, dept})\mathbf{RR^*_1}(\text{sex, age, year, dept})$

$\boldsymbol{\lambda}_2(\text{sex, age, year, dept}) = \boldsymbol{\lambda}_{\mathbf{ref}}(\text{sex, age, year, dept})\mathbf{RR^*_2}(\text{sex, age, year, dept})$

$\boldsymbol{\lambda}_3(\text{sex, age, year, dept}) = \boldsymbol{\lambda}_{\mathbf{ref}}(\text{sex, age, year, dept})\mathbf{RR^*_3}(\text{sex, age, year, dept})$

$\boldsymbol{\lambda}_4(\text{sex, age, year, dept}) = \boldsymbol{\lambda}_{\mathbf{ref}}(\text{sex, age, year, dept})\mathbf{RR^*_4}(\text{sex, age, year, dept})$

$\boldsymbol{\lambda}_5(\text{sex, age, year, dept}) = \boldsymbol{\lambda}_{\mathbf{ref}}(\text{sex, age, year, dept})\mathbf{RR^*_5}(\text{sex, age, year, dept})$

In this final step, the simulated French LT stratified by sex, age, year, French *Département* and deprivation is obtained.

This procedure was reproduced with both available external sources considered in the present study (England LT and EDP), conducting in the two sets of deprivation-specific simulated French LT.

REFERENCES

1. Tron L, Belot A, Fauvernier M, et al. Socioeconomic environment and disparities in cancer survival for 19 solid tumor sites: An analysis of the French Network of Cancer Registries (FRANCIM) data. *Int J Cancer*. 03 2019;144(6):1262-1274. doi:10.1002/ijc.31951
2. Guillaume E, Pornet C, Dejardin O, et al. Development of a cross-cultural deprivation index in five European countries. *J Epidemiol Community Health*. May 2016;70(5):493-9. doi:10.1136/jech-2015-205729
3. Pornet C, Delpierre C, Dejardin O, et al. Construction of an adaptable European transnational ecological deprivation index: the French version. *J Epidemiol Community Health*. Nov 2012;66(11):982-9. doi:10.1136/jech-2011-200311
