# Supplementary material for: Is the Social Gradient in Net Survival Observed in France the Result of Inequalities in Cancer-Specific Mortality or Inequalities in General Mortality?
Source: Cancers (Basel). 2023 Jan 20;15(3):659. doi: 10.3390/cancers15030659 (PMC9913401; doi:10.3390/cancers15030659)
Supplement: Supplementary file 1 [file cancers-15-00659-s001.zip › Supplementary Material_revised.pdf]

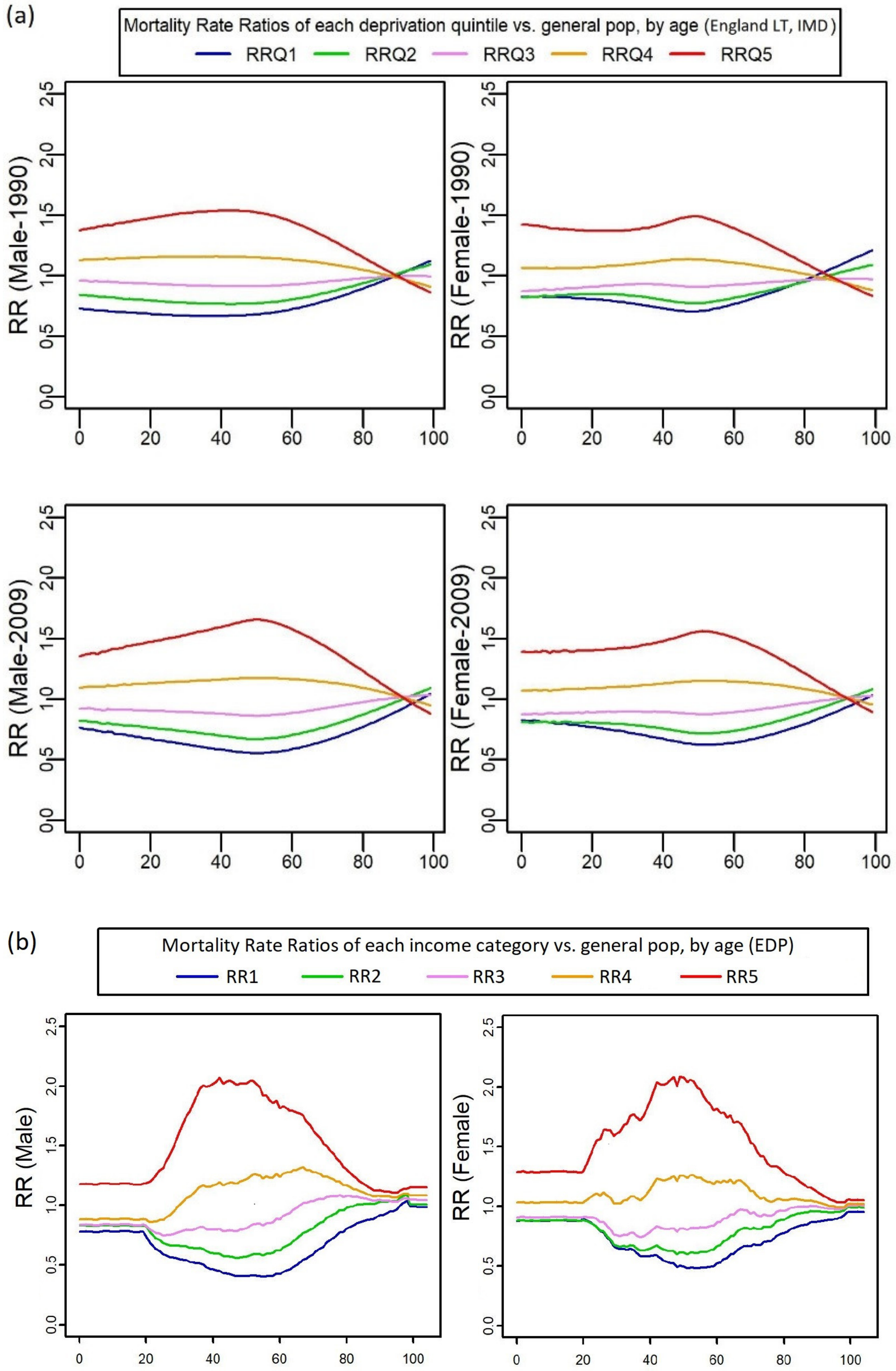

Supplementary Figure S1. Mortality rate ratios (RR) according to (a) quintiles of IMD in the England LT (examples for 1990 and 2009 since all the years were available) and (b) the 5 categories of income in the EDP (for the period 2012-2016).

EDP: French permanent demographic sample [*Echantillon Démographique Permanent]*; IMD : Indice of Multiple Deprivation; LT: life tables; RR: mortality Rate Ratios.

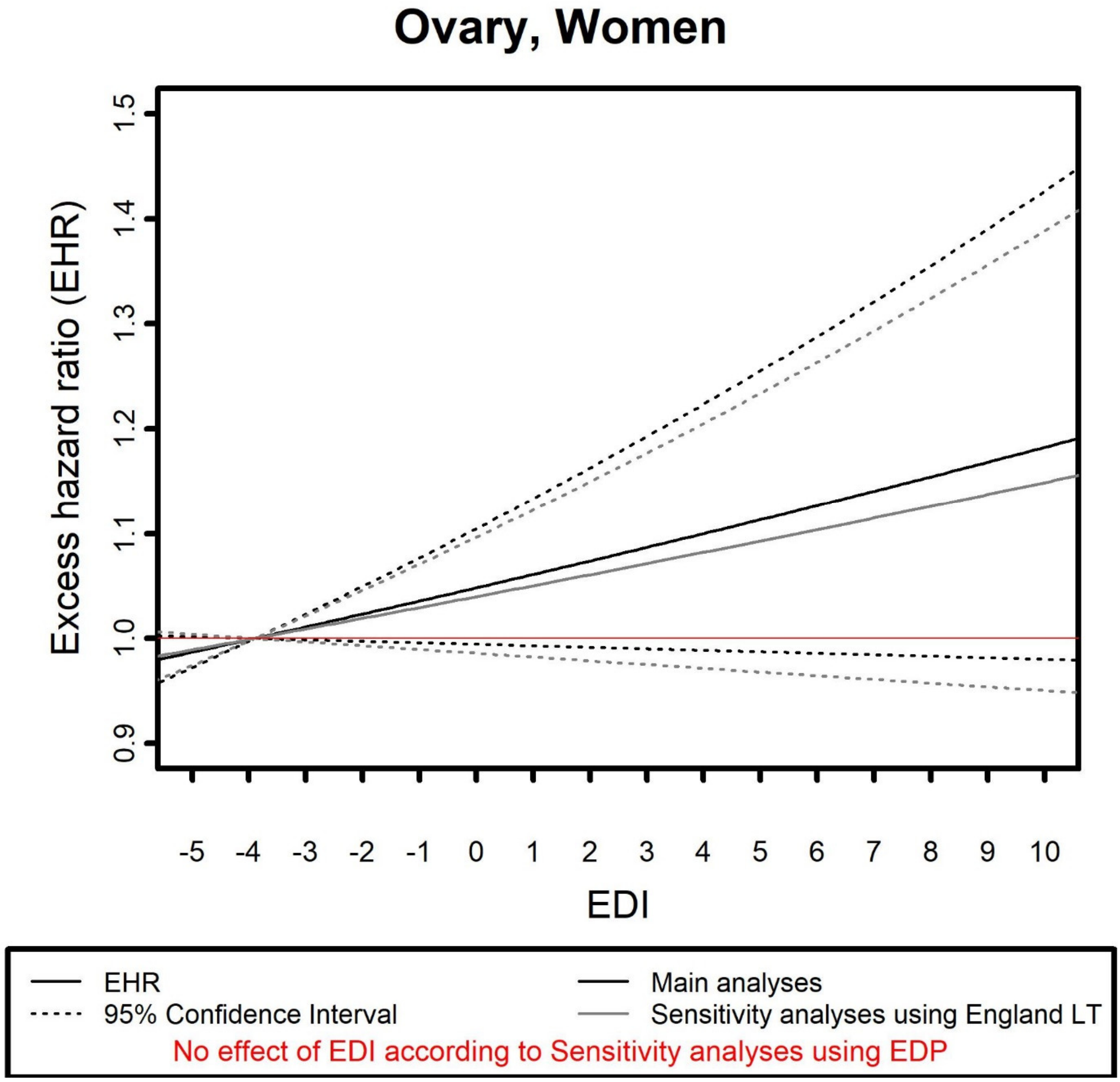

Supplementary Figure S2. Excess mortality hazard ratio (EHR) as a function of deprivation (EDI) according to main analyses and sensitivity analyses using England life tables, for ovary cancer (discordant results between main and sensitivity analyses).

EDI: European Deprivation Index; EDP: French permanent demographic sample [*Echantillon Démographique Permanent]*; EHR: Excess mortality Hazard Ratio; LT: life tables; p90, p10: 90th and 10th percentile of the national distribution of EDI.
